# Advances in drug-induced liver injury research: in vitro models, mechanisms, omics and gene modulation techniques

**DOI:** 10.1186/s13578-024-01317-2

**Published:** 2024-11-02

**Authors:** Kaidi Guo, Twan van den Beucken

**Affiliations:** Department of Toxicogenomics, GROW - Research Institute for Oncology & Reproduction, Maastricht University, Maastricht, 6200 MD The Netherlands

**Keywords:** Drug-induced liver injury, Preclinical models, Mitochondrial dysfunction, Omics, Personalized medicine

## Abstract

Drug-induced liver injury (DILI) refers to drug-mediated damage to the structure and function of the liver, ranging from mild elevation of liver enzymes to severe hepatic insufficiency, and in some cases, progressing to liver failure. The mechanisms and clinical symptoms of DILI are diverse due to the varying combination of drugs, making clinical treatment and prevention complex. DILI has significant public health implications and is the primary reason for post-marketing drug withdrawals. The search for reliable preclinical models and validated biomarkers to predict and investigate DILI can contribute to a more comprehensive understanding of adverse effects and drug safety. In this review, we examine the progress of research on DILI, enumerate in vitro models with potential benefits, and highlight cellular molecular perturbations that may serve as biomarkers. Additionally, we discuss omics approaches frequently used to gather comprehensive datasets on molecular events in response to drug exposure. Finally, three commonly used gene modulation techniques are described, highlighting their application in identifying causal relationships in DILI. Altogether, this review provides a thorough overview of ongoing work and approaches in the field of DILI.

## Introduction

Drug-induced liver injury (DILI) refers to a series of adverse reactions caused by various pharmaceutical drugs (including prescription and over-the-counter drugs), herbal, and dietary supplements through multiple mechanisms [1]. DILI is a significant global health concern, with estimated annual incidence rates ranging from 1.3 to 19.1 per 100,000 individuals worldwide, and the prevalence and etiology vary geographically [2]. DILI typically occurs after a period of several days to months following drug exposure, whereas liver injury resulting from drug overdose presents within hours to days. The symptoms of DILI or hepatotoxicity vary among individuals. In some mild or chronic cases, patients may not experience any symptoms of liver damage, while frequent episodes of acute liver injury are responsible for the development of acute liver failure [3].

DILI is a safety concern that leads to the termination of clinical drug development programs and is the primary reason for drug withdrawal post-marketing [4, 5]. DILI may remain undetected prior to drug approval, and preclinical safety data is insufficient for early prediction in humans [6]. Furthermore, diagnosing DILI is challenging and relies on obtaining a comprehensive medication history from the patient to rule out other potential causes of liver injury [7].

The assessment of DILI on an international scale commonly involves the use of CIOMS Roussel-Uclaf Causality Assessment Method (RUCAM) scale, the Maria & Victorino System of Causality Assessment, and the Clinical Diagnostic Scale (CDS) [8]. However, the scales are prone to instability issues and do not account for all the risk factors in patients. Therefore, it is imperative to obtain dependable and efficacious biomarkers that facilitate the prediction or diagnosis of DILI.

## In vitro models to assess DILI

To acquire useful preclinical data DILI screening should be performed in a robust and physiologically relevant model system. Traditional animal models have limited clinical relevance and, more importantly, there are significant species differences in drug metabolism [9–12]. Two-dimensional (2D) or three-dimensional (3D) cell models are currently the most widely used tools for developing preclinical drugs and exploring the drug-specific risks of DILI. The application of in vitro models also better comply with the 3R principles. This chapter presents a detailed overview of commonly applied and potentially promising in vitro systems, along with their respective advantages and disadvantages.

### HepG2 cells

HepG2 is a hepatocellular carcinoma cell line with high morphology and positive differentiation. This cell line has been generated in the 1970s and has been used in over 34,021 biomedical studies up to date. Yet, there are two important concerns regarding this model. First, although drug metabolism is clearly active in HepG2 cells, drug metabolizing enzymes are not expressed at similar levels compared to primary human hepatocytes (PHHs). This is particularly relevant in studies trying to understand correlations between drug dose and adverse outcome. Therefore, this model is not suitable for determining parameters like lowest-observed-adverse-effect-level (LOAEL) or acceptable daily exposure. The second caveat of HepG2 is the unstable, aneuploid genome, which consists of 50–60 chromosomes. This makes adequate interpretation of functional genomics data generated with HepG2 extremely difficult. Despite these two critical shortcomings, the model itself or data previously generate using this model is still being used in large EU projects EU-ToxRisk, RISK-HUNT3R and ONTOX.

### HepaRG cells

The HepaRG cell line was developed as a more biological relevant model for toxicological studies. When fully differentiated, they resemble mature hepatocytes and retain typical liver function [13, 14]. HepaRG cells have unique biological characteristics, initially appearing as a narrow and long undifferentiated morphology while expressing biological markers of hepatic progenitor cells at low density. After one week, these cells differentiate into distinct hepatocyte morphology and bile duct-like structures.

In comparison to HepG2, HepaRG cells exhibit elevated levels of CYP activity, including CYP1A2, CYP2B6 and CYP2C9, and express phase II enzymes and membrane transporters when exposed to 2% dimethyl sulfoxide (DMSO) [15]. Simultaneously, differentiated cells fully express several drug-related nuclear receptors (NRs), such as the constitutive androstane receptor (CAR), pregnane X receptor (PXR) [16, 17], and three isoforms (α, γ and β/δ) of peroxisome proliferator-activated receptor (PPAR) [17, 18].

Both transcript levels and activity of drug-metabolizing enzymes in HepaRG cells mimic those of primary human hepatocytes (PHHs). HepaRG are more economical, convenient, and show less variation compared to commercial PHHs [19]. HepaRG is suitable for measuring toxic responses based on xenobiotic metabolism and are a superior substitute for HepG2 in DILI investigations. Additionally, HepaRG can also be used for in vitro studies of targeted drugs in cholestasis and steatosis [20–22].

However, HepaRG also has drawbacks. High concentrations of DMSO (e.g., 2% DMSO) used for HepaRG cell differentiation may affect the determination of hepatotoxicity results. Rodrigues et al. retained 2% DMSO to maximize the metabolic activity of differentiated HepaRG cells. But they also acknowledged that while DMSO ensures maximal differentiation stages of cells and maintains high expression of CYP, it is also possible to influence the effects of the compounds analyzed [23]. Verheijen et al. emphasized the ability of DMSO to induce alterations in cellular processes in hepatocytes, and suggested that the use of DMSO should be avoided as much as possible. Even though it is indispensable in biotechnological applications, its concentration should be kept as low as possible [24]. The good news is that undifferentiated HepaRG cells embedded in a collagen matrix rapidly organize into differentiated HepaRG polarized hollow spheres without DMSO, and this model is particularly suitable for evaluating the effects of drugs on cholestasis and steatosis after long-term treatment [25].

Furthermore, Rodrigues et al. also mentioned the low levels of certain enzymes involved in drug detoxification and biotransformation in HepaRG cells, such as CYP2D6 and CYP2E1 [23]. The issue with CYP2E1 is gradually being addressed [26].

### Primary human hepatocytes

During preclinical drug development, in vitro models established based on PHHs are considered the gold standard for evaluating liver drug toxicity. They offer great advantages in the pharmacy realm. PHHs retain the complete morphology of human hepatocytes, maintain consistency with the in vivo environment after isolation, and have biological enzyme content and cofactor levels that mimic typical physiological concentrations [27].

PHHs exhibit higher metabolic activity compared to any other hepatocyte model and best represent in vivo liver conditions. In contrast to monolayer cultures, PHHs in suspension have been observed to not only more accurately estimate clearance rates within a very short period but also retain robust liver functionality, thus providing a more authentic reflection of DILI scenarios [28]. However, since liver toxicity typically manifests several hours later, PHHs in suspension cannot maintain viability within the necessary timeframe. PHHs are capable of sustaining functional activity for 24–72 h, usually requiring adhesion to collagen, with monolayer cells showing higher enzyme stability [29, 30]. However, after several days of conventional 2D culture, cells gradually lose both their morphology and functionality [31–33].

Many efforts have focused on prolonging the hepatic phenotype of PHHs in cell culture. It is generally recognized that culturing PHHS in a collagen sandwich culture can sustain the secretion of plasma proteins, enhance the gene expression of energy metabolism, and partially promote CYPase activity [34–36]. Additionally, Upcyte technology has been applied to establish human hepatocytes with proliferative properties, which has been utilized to clinically assess acute and repeated dose hepatotoxicity [37, 38].

In 2019, Xiang et al. found that PHHs showed an average 10-fold down-regulation of multiple important liver specific genes after 24 h of in vitro culture. The main components of the transforming growth factor beta (TGF-β) signaling pathway and epithelial-mesenchymal transition (EMT) inducers were up-regulated, leading to PHHs dysfunction. In order to maintain the correct hepatocyte phenotype during in vitro culturing, five chemicals were employed that work synergistically with each other: FSK (an adenylate cyclase activator), SB43 (a TGF-β inhibitor), DAPT (a Notch inhibitor), IWP2 (a Wnt inhibitor) and LDN193189 (a BMP inhibitor). This protocol effectively maintains the expression of functional genes and key transcription factors of PHHs (such as CEBPA, CREBH, HNF4A, PXR, CAR, CYP3A4, CYP2D6, ARG, UGT1A6, and NTCP), while inhibiting the expression of mesenchymal marker genes [39]. This is a positive finding that has inspired some researchers to apply this advanced and efficient method to explore the mechanisms of HBV antiviral treatment, and lipid and glucose metabolism [40, 41]. Zhong et al. even confirmed that single-layer 5 C-PHHs perform as well as 3D-PHHs and can be more conveniently used for preclinical drug safety assessment [42].

Another limitation of PHHs is their restricted availability, as this is dependent on scarce organ donations and patient biopsies. In addition, the isolated PHHs are not naïve in terms of exposure and could already be in an unhealthy state.

### Hepatocyte-like cells derived from induced pluripotent stem cells

The development of pluripotent stem cell technology nearly two decades ago, has made it possible to generate an unlimited source of patient-derived cell models for disease modeling [43]. Ever since, various specific cell types have been generated using sophisticated differentiation strategies including hepatocyte-like cells [44, 45]. iPSCs are similar to human embryonic stem cells (hESCs) in terms of cell morphology, gene expression, epidermal modification, and proliferation and differentiation capabilities [46]. Importantly, there are less serious ethical controversies related to be used of iPSCs versus hESCs [47, 48].

iPSCs progressively differentiate into hepatocyte-like cells (HLCs) through endoderm induction, liver specificity and maturation. This is achieved by chronologically adding Activin A, Wnt3, bone morphogenetic protein (BMP) and fibroblast growth factor (FGF), dexamethasone and hepatocyte growth factor (HGF) as supplements [49, 50]. iPSC-derived HLCs exhibit several liver functions, including of serum protein secretion, urea synthesis, glycogen storage, and CYP enzyme expression [51–53].

As an abundant source of human hepatocytes, iPSC-HLCs exhibit comparable phase II enzyme expression to HepG2, and a similar metabolite profile to HepaRG [54, 55]. Furthermore, the sensitivity of HLCs to various hepatotoxic compounds and the response to AHR agonists are similar to those of PHHs [56–59]. As a result, these cells are currently widely used for high-throughput hepatotoxicity screening [60–62] and even transcriptomic compound toxicity research [63, 64]. HLCs also serve as tools for repeated dose testing of single or combination drugs, such as rifampicin and/or cyclosporine A, amiodarone and troglitazone [65, 66].

One major challenge that applies to all iPSC-derived cell models is to resolve the immature state of the final cell type. iPSC-derived cells often resemble fetal rather than adult cells in terms of their epigenome, gene expression profiles and phenotype [67, 68]. To address this issue, several studies have explored strategies to significantly improve the maturity of HLCs [57, 69]. HLCs can be transferred to 3D scaffolds permitting a more physiological arrangements and interaction of cells. The details of culturing iPSC-HLCs based on 3D scaffold structure will be explained in Sect. Liver spheroids. The overexpression of liver-enriched transcription factors (HNF4α and HNF1α) and forkhead box (FOXa2 and FOXa3) was discovered to improve differentiation efficiency and promote iPSC-HLCs maturation [70], which was successfully used to assess drug-induced cytotoxicity [71].

Overall, iPSCs-HLCs are capable of facilitating compound optimization, precision medicine, and drug screening, but protocols for standardizing these cells still need improvement. Furthermore, the current price of commercially available iPSC-HLCs is 30-50% higher than that of PHHs [72], which hinders their widespread application.

### Multicellular co-cultures

The liver is composed of various cells, including hepatocytes, which are the most abundant and mainly responsible for metabolism, biliary epithelial cells, hepatic stellate cells (HSCs) that store vitamin A, Kupffer cells (KCs) that are resident macrophages, and hepatic sinusoidal endothelial cells. They coordinately regulate liver function in multiple microenvironments [73]. DILI may be caused by interactions between different types of liver cells. Therefore, a more comprehensive model should incorporate multiple cell types. Substantial improvements in in vitro models can be achieved through developing co-cultures composed of liver parenchymal cells (PCs) and non-parenchymal cells (NPCs). Applying such strategies leads to functional properties that mimic the liver microenvironment more closely and thus the in vivo conditions [74, 75].

Due to the release of inflammatory mediators and growth factors activated by drugs, HSCs and KCs play crucial roles in regulating DILI. Therefore, most co-culture systems for DILI involve combinations of PHHs and HSCs and/or KCs [76–79]. Compared to monolayer hepatocyte cultures, combined PHHs have been shown to have longer lifespans and better functionality, making them more suitable for long-term studies involving repeated drug administration while retaining responsiveness to inflammatory stimuli [80, 81]. Bell et al. identified the optimal ratio of PHHs to NPCs as 2:1, and found supplementation of NPCs in PHH spheroids mitigated the toxicity of acetaminophen (APAP). Meanwhile, spheroids exhibited lower expression levels of several CYP450 enzymes involved in APAP bioactivation (e.g., CYP2E1, CYP3A4, and CYP1A2), as well as higher levels of miR-382 and miR-155, which have potential roles in liver regeneration and inflammation. They emphasized the potential value of adding NPCs to PHH spheroids for mechanistic studies of APAP-induced immune and steatosis responses [82]. Additionally, besides PHHs and NPCs, Nguyen et al. established a co-culture of human-derived hepatocytes and KCs, finding that compared to single cultures, the co-cultures increased the release of pro-inflammatory cytokines under IL-1β mediation, upregulated acute-phase proteins, and inhibited metabolic enzymes and transporter proteins to evaluate the indirect effects of cytokines on hepatocytes [75].

Micropatterned co-culture (MPCC) technology allows control of cell arrangement in the co-culture platform, which increases the interaction area between cells while providing high-resolution observation of cell behavior and signaling [83]. MPCC created from multiple donors reflects high sensitivity and near-perfect specificity to hepatotoxic drugs, allowing improved prediction of DILI without sacrificing specificity [57, 83]. Currently, MPCCs generated from PHHs and mouse fibroblasts have been utilized to further investigate the relationship between the inhibitory potential of the bile salt export pump (BSEP) and the risk of DILI [84], as well as to simulate the environment of non-alcoholic fatty liver disease (NAFLD) and evaluate valproic acid (VPA)-induced hepatic steatosis [85].

In comparison to 2D monolayer co-culture, most publications prefer to implement co-culture in 3D models. Therefore, this term will be frequently referenced in the subsequent 3D culture section.

### 2D versus 3D cultures

#### 2D cultures

Conventional 2D culture system has been practiced widely for many years. The standard method involves spreading a monolayer of hepatocytes on an ECM-pretreated culture dish to allow them to adhere and diffuse. This modality is inexpensive and well-established, making it easier to observe and measure cells while maintaining critical cellular functions over a short period of time. However, hepatocytes in this system gradually undergo morphological and structural changes over time and rapidly lose function due to epithelial-mesenchymal transition.

As previously stated, the adhesion of hepatocytes between two layers of ECM to form a sandwich structure of cell-cell and cell-matrix interactions is a promising method for drug toxicity screening. Sandwich cultures are able to preserve cell viability, decelerate functional decline, stabilize basic DMETs and related gene expression [86, 87], and enhance the secretion of organic compounds such as urea and albumin for a prolonged duration [88, 89] compared to cells seeded on top of a collagen matrix. Recent studies have utilized more advanced biomaterials, such as artificial materials with hydrogel properties, as substitutes for collagen and Matrigel to imitate the liver microenvironment [90], or attempted to restructure or purify ECM to improve hepatocyte attachment and viability [91].

#### 3D cultures

3D cultures enable cells to better interact with their surrounding environment compared to 2D cultures. It is believed that therefore 3D cultures more accurately represent the real cell microenvironment, simulates the structure and function of tissues, and reflects differences in drug treatment. 3D configured cells exhibit complex cell-cell, cell-matrix, and cell-nutrient interactions, as well as more physiologically relevant properties, such as integrin ligation, cell contraction, and intracellular signaling [92, 93]. This section provides an explanation of several commonly used 3D liver culture models.

##### Liver spheroids

Hepatocytes are capable of self-assembling and reconstituting cell contacts to form non-adherent aggregates known as spheroids. Spheroids comprise three concentric zones: an outer zone composed of less mature or undifferentiated cells, an intermediate zone where cells transition from an undifferentiated to a differentiated state, and a central zone consisting of mature or differentiated cells. These cellular layers are defined by microenvironmental conditions and signaling molecules that vary from the periphery to the center of the spheroid. The simplest protocol for culturing spheroids involves seeding hepatocytes in ultra-low attachment vessels and centrifuging them to establish spheroids through their spontaneous self-aggregation [33]. Another option is to accumulate droplets of suspended cells into microtissues under the influence of gravity. The spheroids can be harvested for direct histological analysis or transferred to non-adherent well plates for long-term culture or drug screening [94]. The spheroids established in both cases sustain liver polarity and expression of typical markers for at least five weeks [95]. Liver spheroids are composed of one or various cell types, including immortalized hepatocytes, PHHs or iPSC-HLCs with or without NPCs. Although experimental conditions using immortalized hepatocyte spheroids might be more difficult to control. As these cells continue to proliferate, the spheroid size will increase over time, creating a hypoxic core that eventually becomes necrotic [96].

Spheroids produced through non-scaffold growth methods are typically uniform in both size and shape. In comparison to monolayer cultures, all hepatocyte spheroids exhibit consistently higher levels of albumin and urea secretion, as well as phase I and II metabolic enzyme activities and transport factor intensities [97–99]. HepaRG spheroids display more sensitive cytotoxicity upon repeated exposure to some hepatotoxic drugs, such as APAP, VPA and cyclophosphamide [100]. It is possible that HepG2 spheroids are less sensitive to DILI than HepaRG spheroids due to low or absent expression of several CYP enzymes [101]. However, Basharat et al. suggested that spheroids derived from the modified HepG2, HepG2 (C3A), exhibited greater sensitivity to DILI compared with HepaRG spheroids when exposed to 150 compounds [102]. PHH spheroids offer distinct advantages as a 3D in vitro model of DILI. Bell et al. developed an approach for preserving and extending the viability, transcriptomic, proteomic and metabolomic phenotypes and functions of PHH spheroids in the absence of serum and demonstrated their suitability for detecting long-term DILI [33]. Meanwhile, compared with PHHs monolayer culture, PHHs spheroids show moderate sensitivity and high specificity after treatment with hepatotoxic drugs, and long-term exposure improves the sensitivity of detecting DILI-positive drugs [103, 104].

Scaffold-free 3D spheroids offer benefits in simulating interactions, whereas scaffold-based cultures are relatively more stable and efficient. It is possible that cells encapsulated in a material scaffold adhere and develop steadily because of the physical support provided. Scaffold materials are categorized into natural extracellular matrix (ECM) and synthetic materials with hydrogel properties, such as poly-l-lactic acid and hybrid poly-l-lactic acid/poly. Scaffold-based system appears to be more suitable for iPSCs-derived HLCs. In comparison to traditional 2D culture, 3D scaffolds expedite liver maturation of HLCs and substantially increase signature mRNA levels, irrespective of their biochemical properties [105–108]. Compared to synthetic materials, HLCs differentiated in 3D ECM-based scaffolds exhibit significantly higher expression of mature liver markers and CYP450 enzyme activity, as well as enhanced proliferation ability [108, 109]. Additionally, Parvanak et al. discovered that up-regulation of miR-122 and down-regulation of off-let-7f further improve the differentiation of scaffold-based cultured HLCs without the need for exogenous growth factors. But this approach has not yet been widely adopted [110].

##### Liver organoids

Organoids contain a population of self-renewing stem cells that share a spatial organization similar to that of their corresponding organs and are capable of reproducing partial functions, thereby providing a relevant pathophysiological system [111]. In contrast, spheroids are usually formed by the aggregation of one or more cell types and have a relatively simple structure. Spheroids are typically simpler to construct, while organoids tend to maintain organic complexity and functional architecture [112]. Human liver organoids (HLOs) are ideal in vitro models that can capture the complex interactions between PCs and NPCs within the liver to regulate metabolic activity.

HLOs express phase I and II enzymes for up to three months at levels equivalent to PHHs and human liver [113, 114]. Consequently, HLOs models for investigating and predicting DILI have been developed. Au et al. established organoids using HepG2 and NIH-3T3 cells and introduced the organoids for drug screening (MODS) platform to analyze pathways of cell apoptosis and necrosis induced by APAP [115]. Leite et al. observed fibrotic characteristics in HLOs derived from HepaRG cells and HSCs after repeated exposure to allylalcohol and methotrexate for two weeks, and these features include HSCs activation, collagen secretion and deposition. Additionally, based on the identification of these organoids, it was found that APAP is able to indirectly induce HSCs activation, thereby mediating hepatotoxicity. This conclusion was further validated in mice [116].

HLOs generated from iPSCs are more practical for large-scale DILI risk assessment because of their scalability. Zhang’s team demonstrated that iPSCs-HLOs from three different sources exhibit DILI predictive capabilities similar to intact HLOs in high-throughput screening, and observed clear morphological disparities in cells treated with drugs targeting different mechanisms [117]. Additionally, they established the patient-derived liver-on-chip (PaDLOC) system, validating that iPSCs-HLOs combined with PaDLOC show high physiological similarity to the human liver, enabling assessment of more complex DILI mechanisms [117]. The DILI identification method developed using iPSCs-HLOs displayed high sensitivity (88.7%) and specificity (88.9%) for 238 marketed drugs at four different concentrations [61]. Addressing the issue of limited expansion and further differentiation of iPSCs-HLOs, Mun et al. devised an efficient and reproducible strategy for generating iPSCs-HLOs [118]. Additionally, they verified the maturity of these organoids through comprehensive transcriptomic analysis and performance assays, confirming their heightened susceptibility to clinical drugs capable of inducing hepatotoxicity and steatosis [118].

Future advancements may position HLOs as a standard in vitro model for compound toxicity screening. Some drawbacks of HLOs, including limited maturity and functionality, as well as challenges in controlling heterogeneity [119], which are indeed also present in liver organoids, are expected to be gradually overcome.

##### 3D bioprinted liver

3D bioprinting is the utilization of 3D printing technique to integrate cells, growth factors, and/or biomaterials, aiming to fabricate biomedical components. The application of this technology to study liver disease emerged only a few years ago. 3D bioprinting facilitates the spatially coordinated growth of almost all hepatocytes, encompassing HepG2 [120, 121], HepaRG [122], PHHs [123, 124] and iPSCs-HLCs [53], and less commonly used hESCs- HLCs [125] and iPSCs-derived liver progenitor cells (HPCs) [126]. All of them retain stable functionality and viability in long-term culture.

Liver tissues generated by 3D bioprinting have demonstrated superior sensitivity and accuracy in detecting DILI compared to 2D cultures and 3D spheroids for a selection of hepatotoxic compounds [53, 122, 123]. Moreover, liver bioprinting effectively replicates DILI scenarios and distinguishes highly homologous compounds, such as trovafloxacin and levofloxacin, both belonging to the quinolone class [124]. Interestingly, an emerging model named exVive3D bioprinted liver is being exploited as the first commercially available human liver tissue to evaluate drug hepatotoxicity [127]. ExVive3D liver tissue closely resembles native liver tissue with intercellular connections, microvascular structures, and cellular compartments. Damage caused by APAP insult mirrors that observed in liver specimens from patients overtreated [128]. In brief, it is possible that the remarkable biological properties of 3D bioprinted liver models improve the assessment of potential clinical DILI risk to some extent.

##### Liver-on-a-chip

Microfluidic culture devices are a branch of microfluidic technology, and organs-on-chips represent a specialized subtype of microfluidic chips [129]. Organs-on-chips incorporate physiologically relevant fluid shear stress and cyclic strain, analyze organ-specific responses to antigenic invasion, as well as more e precisely adjust environmental conditions based on the cultured cell types [130].

Liver-on-a-chip has emerged in recent years, resulting in limited literature on DILI. Bhise et al. encapsulated HepG2/C3A cells within hydrogels to construct liver spheroids, which were then arranged in microfluidic chips. After one month, they found that metabolic activity and four biomarkers of the chip significantly decreased over time post-APAP treatment. This suggests the potential of liver-on-a-chip as a complementary tool for assessing acute drug toxicity [131]. Zuchowska’s team cultivated HepG2 spheroids in a microfluidic system and observed their response to selected concentrations of 5-fluorouracil (5-FU), revealing the decrease in 5-FU resistance with increasing spheroid diameter [132]. Moreover, a large-scale screening of 122 clinically hepatotoxic drugs indicated that the 3D PHHs model integrated into a biomimetic chip displayed greater sensitivity to DILI compared to 2D cultures [133].

Microphysiological system (MPS) is defined as a novel microfluidic platform that reconstruct the characteristics of tissue microenvironments [134]. This sophisticated platform has the capability to enhance cell differentiation and maturation and extend enzyme function and albumin production for at least one month after isolation or inoculation [135]. Hepatic MPS successfully replicate the hepatotoxic effects of trovafloxacin across various batches. Furthermore, owing to the sustained activity of CYP3A4 and albumin secretion over an extended duration, liver cell function within MPS remains more stable [136].

Liver-on-a-chip demonstrates minimal loss of specific functions and viability, while enabling precise control of the local environment and the simulation of human organs, surpassing other 3D technologies [135, 137]. Furthermore, compared to static models, perfusion of cultures allows for continuous nutrient exchange and oxygen delivery, which enhance and sustain liver-specific functions [138]. We look forward to more evidence from liver-on-a-chip technology to further explore DILI in the future.

## Cellular responses as targets for DILI

**Fig. 1:**
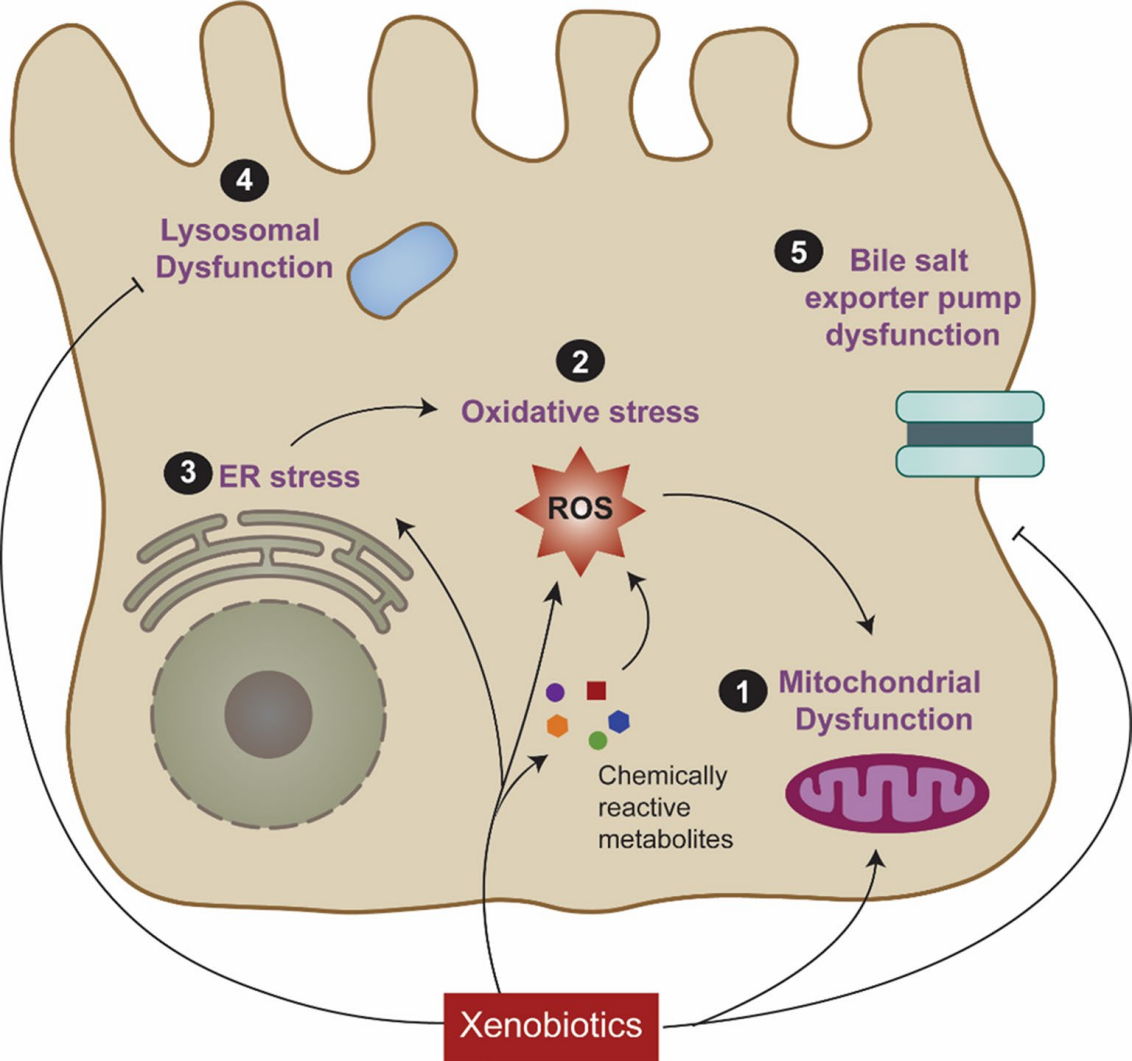
A reductionists view of DILI. Xenobiotic substances can trigger a range of intracellular disturbances linked to DILI risk, including (**1**) mitochondrial dysfunction, (**2**) production of reactive metabolites and oxidative stress, (**3**) ER stress, (**4**) Lysosomal impairment, and (**5**) inhibition of biliary efflux

The U.S. Food and Drug Administration (FDA) and the European Medicines Agency (EMA) have both established industry guidelines for premarketing clinical and/or nonclinical evaluation of DILI. Nonetheless, there has been limited progress in confronting the regulatory obstacles created by the adverse drug effects. Biomarkers such as alanine aminotransferase (ALT), aspartate aminotransferase (AST), alkaline phosphatase (ALP) and bilirubin, have been utilized for over four decades as indicators of liver drug safety [139]. The insufficiency of these markers has been recognized in FDA guidelines. In the following section, the most prominent cellular mechanisms underlying DILI will be discussed (Fig. 1).

### Mitochondrial dysfunction

Mitochondria are vital organelles in eukaryotic cells, primarily responsible for generating adenosine triphosphate (ATP) to meet the energy demands for cell survival. They also play an indispensable role in liver synthetic metabolism and catabolism [140]. Defects or alterations in mitochondrial activity can result in mitochondrial dysfunction (MDF), leading to the onset of various diseases.

The concept that drugs may interfere with mitochondrial function as a mechanism of hepatotoxicity originates from 1995, when clinical trials documented symptoms of mitochondrial damage in patients receiving drug therapies [141]. Since then, evidence of adverse events caused by DILI that leads to MDF has continuously expanded. In 2021, FDA released a report listing 192 compounds categorized as having the highest risk of DILI, with calculations indicating a higher proportion of drugs capable for triggering mitochondrial toxicity, resulting in severe liver injury in patients [142].

Mitochondrial energy production primarily relies on oxidative phosphorylation (OXPHOS) and the tricarboxylic acid cycle (TCA). Several drugs cause severe ATP depletion by directly harming OXPHOS and electron transport chain (ETC) in the liver, encompassing two procedures. Some non-steroidal anti-inflammatory drugs (NSAIDs) such as ibuprofen and nimesulide, uncouple OXPHOS without inhibiting ETC activity [143, 144], but their effects are relatively mild and may not lead to adverse consequences in vivo. Dual inhibition of OXPHOS and ETC further impair the oxidation process. For instance, amiodarone as an OXPHOS uncoupler restrains ETC and indirectly affect β-oxidation [145]. Unfortunately, the mechanism remains unclear, with evidence suggesting that this interaction appears to depend on drug concentration [146–148].

The mitochondrial permeability transition pore (MPTP) mediates mitochondrial permeability transition (MPT), leading to the loss of structural and functional integrity of the mitochondrial membrane [149]. Mitochondria involved in MPT release pro-apoptotic factors, including Bcl-2 family proteins and cytochrome c [150]. Some drugs trigger uncontrolled opening of MPTP, causing widespread hepatocyte apoptosis and necrosis. High doses of APAP initiate MPT by activating c-Jun N terminal protein kinase (JNK) or other inducers, resulting in irreversible MDF and cell death related to JNK activation [151–155]. Moreover, the JNK pathway can be activated by reactive oxygen species (ROS), leading to the translocation of phosphorylated JNK to the mitochondria, where it interacts with Sab protein, further exacerbating oxidative stress and ROS production [156]. Hepatotoxic drugs that excessively impact oxidative stress [157–159] and ferroptosis [160] have also been shown to interfere with MPT.

Drugs can directly impact β-oxidation by inhibiting the formation of long-chain fatty acids that enter the mitochondria, leading to the accumulation of triglycerides within hepatocytes and consequent steatosis [161]. VPA is capable of consuming free Coenzyme A (CoA) and carnitine palmitoyltransferase (CPT), disrupting β-oxidation and leading to MDF [156, 162]. Tetracycline is believed to interfere with the oxidation process by down-regulating genes involved in β-oxidation and TCA cycle [163, 164]. Additionally, tamoxifen, tetracycline, and fluoroquinolone broad-spectrum antibiotics are hypothesized to disrupt mitochondrial DNA (mtDNA) synthesis by interacting with mitochondrial topoisomerase [147, 165, 166].

The intricate mechanisms of mitochondrial toxicity render it such that distinct endpoints may regulate the same event [167], and the same drug may influence different mitochondrial functions [156, 159, 168]. Additionally, DILI is multifaceted, and solely assessing MDF appears to be inadequate for evaluating the risks of DILI. Therefore, it may be valuable to consider co-predictions for different drugs or targets.

### Chemically reactive metabolites and oxidative stress

Chemically reactive metabolites (CRMs) arise during the drug biotransformation process and induce DILI by binding to essential macromolecules and blocking their function. DMETs serve as the primary hepatic catalysts for drug metabolism and sometimes produce elevated levels of CRMs [169]. The formation of CRMs may result in oxidative stress [170]. Oxidative stress occurs when there is insufficient antioxidant capacity to deplete the excess ROS and other organic free radicals, a condition implicated in DILI [171].

Although hepatocytes possess an antioxidant system composed of molecules such as glutathione (GSH), superoxide dismutase (SOD), glutathione peroxidase (GPx), and catalase (CAT), the metabolism of some drugs consumes this defense capacity, leading to oxidative stress before liver injury occurs [171]. In addition to the aforementioned MDF, excess intake of APAP also accelerates the synthesis of a toxic CRM named N-acetyl-p-benzoquinone imine (NAPQI). Elevated levels of NAPQI deplete GSH, resulting in defective removal of ROS and activation of various complex molecular pathways, thereby triggering liver damage and hepatocyte necrosis [172].

Antipsychotic drugs at abnormal doses also elicit oxidative stress in the liver. Rats treated with fluoxetine showed an increase in overall oxidative status and a reduction in antioxidant capacity in the liver [173, 174], manifested by the deterioration of SOD, GPx, CAT and GSH, coupled with upregulation of pro-apoptotic factors and downregulation of anti-apoptotic factors [175]. Sertraline is able to cause MDF characterized by rapid ATP depletion and induction of MPT, as well as enhance lipid peroxidation and nitric oxide production in the liver [176, 177]. Both chlorpromazine and clozapine have been proved to undergo N-oxidation to form highly active CRM, generate excess ROS, reduce SOD and CAT enzymatic activities and GSH-dependent defense to impair the antioxidant system ultimately [178]. Clozapine even has a higher potential than fluoxetine to induce hepatotoxicity [173].

One challenge encountered in interventions aimed at oxidative stress is the necessity of administering hepatotoxic drugs at concentrations much higher than those in patients, both in in vitro and in vivo research, to permit the accumulation of CRMs or ROS that leads to DILI toxicity. However, this problem is prevalent in preclinical investigations of DILI, and overcoming the obstacle remains an unresolved issue.

### Endoplasmic reticulum stress

The endoplasmic reticulum (ER) is an organelle that plays an essential role in many cellular processes, including protein synthesis and processing, lipid synthesis and calcium storage. Hepatocytes rely heavily on the ER for producing large amounts of secretory proteins such as albumin and alpha-1-antitrypsin. Hence, hepatocytes are enriched in ER and are extremely sensitive to ER perturbation and stress. ER stress initiates an adaptive cellular response known as the unfolded protein response (UPR), which aims to restore ER homeostasis and promote cell survival. Various conditions trigger UPR activation, including alterations in redox status, glycosylation, calcium homeostasis, and the burden of secretory proteins. Accumulating evidence suggests that hepatotoxic drugs may induce ER stress, potentially contributing to DILI.

UPR signaling is mediated by three distinct ER-resident proteins that act as stress sensors: inositol-requiring protein 1 (IRE1), PKR-like ER kinase (PERK) (also known as EIF2AK3) and activate transcription factor 6 (ATF6). In non-fasted mice subjected to repeated pretreatment with VPA followed by high-dose APAP administration, mRNA levels of CCAAT-enhancer-binding protein homologous protein (CHOP), IRE1 splicing, and eIF2α phosphorylation were induced after 4 h. Furthermore, neither VPA nor APAP alone activated UPR markers compared to the control group [179]. Uzi et al. also demonstrated that oral gavage of APAP strongly stimulated the expression of CHOP, Xba1 splicing, and eIF2α phosphorylation [180]. Li et al. found that SPHK1 levels in the liver were significantly elevated following APAP treatment. The phosphorylation of SPHK1 activates the PERK-eIF2α-ATF4 pathway and ATF6 (activating transcription factor 6), induces the production of CHOP, and ultimately promotes ER stress [153]. We hypothesized that high-dose APAP promotes ER stress, with the apoptotic factor CHOP playing a crucial role in APAP-induced ER stress during DILI.

In addition to APAP, trovafloxacin and efavirenz induce hepatocyte injury by triggering ER stress and UPR at clinically relevant concentrations [181, 182]. However, it is worth noting that ER stress or UPR may be late events in DILI, or even secondary consequences of other adverse events such as MDF and oxidative stress [180, 183]. In conclusion, further investigation is needed to determine whether ER stress is a primary mechanism driving DILI.

### Lysosomal dysfunction

Lysosomes contain over 60 different hydrolases, and defects in lysosomal degradation or transport can lead to lysosomal dysfunction [184]. While lysosomes function as degradation centers in various cells, there is a notable lack of articles on lysosomal dysfunction and DILI. Excessive APAP exposure cause lysosomes to become destabilized and release cathepsin B into the cytoplasm, but it does not instigate liver damage [185], although this lysosomal protease was previously identified for contributing significantly to liver injury [186]. The lysosome is the major source of iron, and the destruction of lysosomes by APAP may lead to the release of Fe²⁺ into the cytoplasm, ultimately resulting in hepatocyte death [151]. Diclofenac has been noted to regulate hepatotoxicity by attenuating cathepsin activity and inhibiting autophagic flux [187].

Lysosomal dysfunction is not necessarily a definitive indicator to prevent drug development. Nevertheless, Uzhytchak’s review enumerated certain nanodrugs that could cause adverse effects through lysosomal dysregulation [188], expecting the possibility of further advancements in the field of nanomedicine.

### Alterations of the bile salt export pump

Drug-induced cholestatic liver injury accounts for the majority of DILI reports. The secretion of bile acids (BAs) is related to the function of the hepatobiliary transporter system. When the homeostasis of BAs is destroyed, the concentration of BAs will elevate abnormally and become hypertoxic to hepatocytes. Bile salt export pump (BSEP) is primarily responsible for excreting BAs into bile through an ATP-dependent mechanism.

Impaired functioning of liver transporters is a key pathogenic factor in cholestasis, with BSEP being the most significant among these transporters. Impaired BSEP caused by defective gene regulation may contribute to hereditary or acquired cholestatic diseases [189]. Several drugs have been identified as BSEP inhibitors. Ogimura et al. employed sandwich-cultured rat liver cells to screen 26 hepatotoxic compounds, of which 11 exhibited BA-dependent drug toxicity, with 9 of them being verified as BSEP inhibitors [190]. Garzel et al. also utilized sandwich-cultured PHHs to assess 30 BSEP inhibitors, demonstrating the significant role of BSEP inhibition in drug-induced cholestatic hepatotoxicity [191]. Moreover, they suggested that the transcriptional suppression of BSEP by lopinavir and troglitazone might result from their interaction with the farnesoid X receptor (FXR) [191]. Interestingly, in human hepatocytes, troglitazone and its metabolites competitively inhibited BSEP leading to hepatotoxicity [191, 192], whereas this phenomenon was not observed using rat hepatocytes [193, 194].

Sulindac and bosentan, both withdrawn from the market due to acute-specific DILI, have also been found to suppress BSEP and accumulate intracellular bile salt-independent bile flux [195–197]. Additionally, Guo et al. detected that isoniazid and rifampicin induced DILI through downregulating BSEP, resulting in bile acids accumulation in mice [198].

An interesting observation is that in vitro models appear to be more sensitive than in vivo models in testing the response to BSEP transcription. This difference could be attributed to variances in bile salt composition and compensatory mechanisms present in rodents [193, 194, 196]. Monitoring alterations in BSEP activity are able to provide insights into the occurrence, delayed manifestations, and species variations of DILI, assisting in the evaluation of the hepatotoxic potential of candidate drugs. However, there are also opposing views. For instance, Chan et al. argued against the notion that BSEP alone or in combination with other liver transporters can accurately predict DILI [199]. Therefore, while BSEP is considered a valuable factor in drug-induced cholestatic hepatotoxicity, its impact on other types of DILI requires further verification.

## Omics technologies for investigating DILI

“Omics” is a broad term referring to research fields in the biological sciences that end with “omics”, such as genomics, transcriptomics, proteomics, and metabolomics. The introduction of omics technologies has tremendously accelerated progress in personalized medicine and pharmacological research. It enables the generation of large, complex datasets describing cellular biology at the molecular level. “Omics” approaches can be used for identification or classification of toxic compounds or to generate mechanistic understanding of cellular processes underlying adverse outcomes to those compounds. Therefore, “omics” technologies are very suited to enhance preclinical safety assessment of DILI [200]. In this chapter, we will enumerate the pros and cons of prevalent omics technologies related to DILI.

### Genomics

Genomics, one of the earliest developed omics, focuses on assembling and analyzing the function and structure of entire genome using high-throughput DNA sequencing and bioinformatics [201]. Single nucleotide polymorphisms (SNPs), which involve substitutions of single nucleotides at specific locations in the genome, contribute to the interpretation of susceptibility to xenobiotics.

The susceptibility to DILI primarily originates from SNPs in immune-related genes, such as the human leukocyte antigen (HLA) or cytokine-related genes. HLA alleles have been identified as significant susceptibility factors for several DILI drugs through genome-wide association studies (GWAS), with strong evidence found in drugs like amoxicillin-clavulanate (AC) [202–205], flucloxacillin [206, 207], and lapatinib [208–212]. Moreover, isoniazid, minocycline, terbinafine and nevirapine have also been linked to high-risk HLA haplotypes [213–216]. Cytokines comprise interleukin (IL)-4, IL-10, and tumor necrosis factor (TNF)-α. Patients with diclofenac-hepatotoxicity exhibited a higher frequency of variant alleles IL-10-A627C and IL-4-T590C, with the highest odds ratio observed when both were present simultaneously [217]. Furthermore, TNF-α-G308A has been found to be significantly associated with hepatitis induced by isoniazid and rifampicin [218].

In addition to immune-related SNPs, genetic variants that lead to decreased activity of certain phase II liver enzymes appear to influence the occurrence of DILI, with N-acetyltransferase 2 (NAT2) being the most typical. Patients with NAT2 mutations displayed reduced substrate acetylation and remarkably enhanced sensitivity to liver injury mediated by isoniazid and rifampicin, both of which are NAT2 substrates [219]. Chan et al. also demonstrated a correlation between isoniazid-DILI and slow acetylation variants of NAT2 [220]. Furthermore, individuals carrying double T1-M1 null genotype of glutathione-S-transferase (GST) are at an elevated risk for DILI caused by troglitazone [221], tacrine [222], NSAIDs and AC [223].

However, predicting the ultimate biological action solely through genomic analysis is challenging due to epigenetic, transcriptional, and post-translational DNA transformations. Therefore, genomics could serve as a baseline for providing comprehensive DNA information.

### Transcriptomics

Transcriptomics, the subsequent layer of genomics, primarily focuses on measuring gene expression levels through quantifying mRNA, and other non-coding RNAs. There are two key contemporary technologies in this field: microarrays and RNA sequencing (RNA-seq). The main difference between them is that the former depends on the hybridization of pre-designed labeled probes with target sequences, while the latter allows for direct sequencing of the entire transcriptome [224]. RNA-seq is presently recommended for transcriptional profiling research because of its broader coverage, absence of species limitations, and greater flexibility [225]. Single-cell RNA-seq can even report the transcriptome of human hepatocytes and even the hepatic immune microenvironment [226].

Transcriptomics-driven toxicogenomics has been advocated for precise determination of dose-dependent hepatotoxicity [227]. Based on the concept of big data compression and fusion, it has also been applied to evaluate diverse DILI cell models and identify the operational mechanisms of specific hepatotoxic drugs [228]. Wolters et al. conducted multi-omics analyses and discovered 6 persistent microRNAs associated with drug-induced cholestatic pathway under repeated doses of cyclosporine A [229]. Two years later, they integrated cross-omics perturbation data to expose new insights into mitochondrial-nuclear crosstalk, supporting for the dynamic induction of MDF, steatosis and hepatotoxicity by VPA [230].

Moreover, transcriptomics can also be utilized for screening potential biomarkers for DILI. Transcriptome differential analysis revealed that non-lethal doses of APAP upregulated the expression of Myc, Bag3 and Btc in mouse liver, whereas lethal doses did not, indicating these genes may play crucial roles in the adaptive protective response to DILI [231]. Ward et al. pointed out significant elevation in the expression of hundreds of microRNAs in plasma or serum after APAP administration, preceding ALP, particularly miR-122 and miR-192 [232, 233]. Meanwhile, there was evidence suggesting miR-122, which exhibits liver specificity, could serve as an emerging DILI biomarker [234].

Transcriptomics is more appropriate as an intermediate step, because post-translational modifications impact the protein expression, and facilitate the identification of alterations in genetic expression profile following toxicological drug treatments.

### Proteomics

Proteomics typically involves protein purification and mass spectrometry. It constitutes a crucial follow-up to genomics and transcriptomics in biological systems. Proteomics is inherently more intricate than genomics because the genome of an organism remains largely constant, whereas its proteome varies across cell types and over time. Moreover, the majority of proteins undergo extensive post-translational modifications [235].

Proteomics has been the standard method for measuring elevated liver enzymes in the blood of patients, but they are only detected after injury has occurred, and some drugs increase plasma liver enzymes without causing actual liver damage. By testing ALT, AST and ALP binding to apolipoprotein E, α-trypsin inter-inhibitor, serum amyloid P-component, gelsolin and complement component C7, a diagnostic accuracy of 95% for DILI can be achieved [236]. Proteomics is able to screen out toxin-reactive proteins superior to ALT and AST in patients with APAP or carbon tetrachloride overdose [237]. Furthermore, proteomics quantified alcohol dehydrogenase 1B, which rose extremely in serum samples of APAP-poisoned patients, and subsequently decreased to minimal levels during recovery [238]. It also quantified calmodulin in mouse urine to identify APAP-DILI [239], an approach that was both non-invasive and preceded ALT increase. Patients were considered as positive for diclofenac-induced DILI when the integrin beta 3 expression in whole blood was below the determined threshold of 60%, and the evaluation of this protein was as simple as liver enzymes and focused specifically on diclofenac [240].

It is also possible that proteomics clarifies mechanisms of hepatotoxicity. In the case of the widely known issue of APAP overdose, it offered valuable insight into the necrosis and self-repair profiles of liver proteins [241], and identified the activation of protective signaling pathway against hepatotoxicity [242]. Long-term low-dose exposure to bisphenol A altered the expression of phosphorylated proteins involved in metabolic and antioxidant defense in the liver [243]. Paemanee et al. found that nearly 40% of differential regulatory proteins after nevirapine treatment were mitochondrial proteins, indicating this drug may mediate mitochondrial dysregulation in liver cells [244].

The application of proteomics in drug toxicology holds promising potential, although it may not be as sensitive to individual proteins as immunohistochemical methods like Western blotting or ELISA. Indeed, the techniques for protein isolation and measurement are quite complex and multi-faceted due to the unique properties exhibited by each protein, such as mass, isoelectric point, solubility and stability. Therefore, the design of experiments and interpretation of proteomic data requires a case-by-case strategy.

### Metabolomics

Metabolomics aims to measure, identify and quantify a wide range of small molecules to understand metabolic pathways, biochemical reactions, and the impact of external factors on metabolism. Metabolomics relies on non-invasive nuclear magnetic resonance (NMR) spectroscopy and invasive mass spectrometry and chromatography. Mass spectrometry stands out as a prominent method for most prospective studies as it can efficiently separate and identify individual components, with high sensitivity and suitability for high-throughput screening. The metabolic processes in human body are quite complex, leading to a wide variety of metabolites. There is currently no consensus on which single or combined metabolites are recommended for regular diagnosis and prognosis of DILI.

BAs are synthesized in the liver and accumulate in the blood during liver injury. Consequently, some studies have demonstrated the use of various bile acids in detecting DILI. Glycochenodeoxycholic acid, taurodeoxycholic acid, taurocholic acid and glycocholic acid are universally accepted as biomarkers [245–248]. They are able to distinguish patients with severe and non-severe DILI [246, 249], or be combine with glycerophospholipids to determine the three phenotypes of DILI [250]. Moreover, they are also suitable for accurate classification of DILI in children [251, 252]. In addition to the four common BAs mentioned above, glycodeoxycholic acid and obeticholic acid have also been implicated as potential biomarkers for DILI [252, 253]. Indeed, BAs appear to be more appropriate for diagnosing cholestatic liver injury [254], because as previously explained, the secretion of BAs is a key aspect of the hepatobiliary transporter system.

Liver injury and dysfunction disrupt protein synthesis, leading to elevated concentrations of free amino acids in the liver and blood, with changes in amino acid doses potentially varying according to the severity of liver injury [255]. Chen et al. identified characteristic metabolic fingerprints of chronic DILI-associated liver fibrosis through metabolic profiling. The elevated levels of metabolites such as phenylalanine, tyrosine, tryptophan, arginine, and proline were mainly concentrated in these metabolic fingerprints [247]. Co-elevation of glutamate, alanine, leucine and phenylalanine in the serum may serve as a unique indication for VPA-DILI [256]. Moreover, first-line antituberculosis drugs impact the metabolic pathways of arginine and proline [257]. Varying opinions on the candidate amino acids for APAP-DILI may stem from age restrictions and geographical differences [258, 259] in clinical trials. Notably, the timing of APAP treatment appears to be the sole factor associated with significant increase in ornithine [260].

Lactate participates in multiple metabolic processes within the human body, and the liver is one of the important lactate-metabolizing organs. Impaired liver function has the potential to influence lactate clearance, and excessive glycolysis also cause the hepatocytes to release lactate. The concept of blood lactate as an early predictor of the outcome in APAP-induced acute liver failure was initially suggested in 2002 by Bernal et al., who speculated that APAP had a direct toxicity on cellular respiration [261]. Therefore, elevated lactate levels usually reflect the severity of APAP-DILI in pharmacometabolomics [258, 259, 262], accompanied by obvious dysregulation of OXPHOS or mitochondrial function [263].

Furthermore, there are possible auxiliary metabolic biomarkers for DILI. Uric acid [246, 256, 257] and hippurate [258, 259], which are the final products of purine metabolism, often emerge in urinary metabolomics. Phospholipids, crucial components of cell membranes, can be perturbed by drug-triggered events such as MDF or oxidative stress [247, 256, 264].

Toxicometabolomics provides a non-invasive method for sample collection, with urine and plasma endogenous metabolites proving to be more sensitive than serum biochemical parameters [259]. Furthermore, it complements a comprehensive understanding on biological systems. In summary, the effective screening of individual-specific DILI biomarkers and gaining insights into the interactions between drugs and biological processes can be achieved by analyzing multiple types of biomolecules through a multi-omics strategy.

## Functional genomics by perturbation

Gene modulation techniques, such as CRISPR/Cas9, RNA interference (RNAi), and antisense oligonucleotides, have become powerful tools for generating mechanistic insights into DILI. These technologies enable precise manipulation of gene expression, allowing researchers to study the functional roles of specific genes involved in DILI mechanisms. These approaches complement traditional “omics” studies by enabling experimental validation of cause-effect relationships, providing deeper mechanistic insights into the cellular processes affected by toxic compounds. They also allow for the investigation of genetic susceptibility factors that may predispose individuals to DILI, making them particularly valuable for advancing personalized medicine and improving preclinical safety assessments. In this section, we will explore the advantages and limitations of widely used gene modulation techniques and their applications in DILI research.

Gene knockdown and knockout are two essential techniques for studying causal events in regulatory networks. Gene knockdown typically involves introducing siRNA, shRNA or other RNA interference tools to influence the translation or stability of target mRNA without entirely eliminating its function. Gene knockout, on the other hand, involves implementing gene-editing technology to completely abolish the function of the target gene, and CRISPR-Cas9 system is the most commonly used method. These fundamental molecular biology tools play an important role in validating potential cellular mechanisms related to DILI that have been identified using “omic” technologies.

### siRNA

Small interfering RNA (siRNA) is a short double-stranded RNA molecule capable of causing degradation or translation inhibition of mRNA by complementary pairing with the mRNA of a target gene [265]. It is possible to achieve short-term temporary gene silencing, with its influence gradually diminishing over time.

Although the application of siRNA for acute gene silencing to create liver disease models began in the early 2000s, the field of DILI has only gained momentum in animal and cell cultures in the last decade. In mice, siRNA targeting specific genes has been used to explore the protection of signaling pathways, particularly JNK, on ibuprofen-induced acute liver injury [266], as well as to assess the role of cyclin M4, which is involved in magnesium homeostasis in the ER during APAP hepatotoxicity [267]. Furthermore, it is also possible to reveal the mechanism of BAs and leukotrienes in APAP-DILI based on silenced G protein-coupled receptor [268].

In DILI studies, Nrf2-deficient siRNAs are frequently used in in vitro models. Kale et al. used siRNA to silence Nrf2 in human liver microsomes and found Nrf2 knockdown did not lead to elevated serum ALT activity or any noticeable histopathological indications of liver damage upon exposure to nimesulide. Their findings indicate that nimesulide is metabolically activated by CYP2C into protein-reactive electrophilic intermediates, which can activate the Nrf2 pathway even at non-toxic exposure levels [269]. Downregulation of NRF2 in PHHs and HepG2 cells was regarded as tool to assess the toxic potential of positive NRF2 signaling [270], characterize the impact of diclofenac and omeprazole on the Nrf2 pathway [271], and elucidate Nrf2-mediated adaptive responses to mitigate rifampicin-induced DILI [272]. This is possibly due to Nrf2, a transcription factor, playing a crucial role in regulating the intracellular antioxidant defense system and drug detoxification pathways [273]. In addition, siRNA-mediated interference in HepG2 and L02 cells provided a clear understanding of the mechanisms underlying the diclofenac- and TNF-α-induced apoptotic crosstalk [274], as well as the alleviation of cell damage in hepatocytes [275].

### shRNA

Short hairpin RNA(shRNA) is a synthetic small RNA molecule, similar in principle to siRNA, which is typically utilized in gene expression vectors to achieve stable and sustained gene silencing within cells. Although siRNA and shRNA perform comparable roles, they are distinct molecules with potentially different mechanisms of action and RNA interference pathways [276].

shRNA technology has been integrated into several liver injury models, including those for hepatitis B virus infection [277], cholestatic liver disease [278], and alcoholic liver disease [279]. Rats carrying shRNA against γ-glutamylcysteine synthetase were utilized in acute or subacute toxicity trials involving diclofenac and flutamide [280]. Additionally, mice with suppressed Grsf1 expression in the oxidative stress pathway were employed to determine the potential mechanisms underlying DILI caused by anti-tuberculosis drugs [281].

Although instances of shRNA serving as a tool to mimic DILI are scarce, extensive research has regarded shRNA as a lentiviral vector to silence specific genes in liver cell models, particularly PHHs. Amet et al. downregulated BST-2 in PHHs using shRNA and found a significant reduction in IFN-mediated antiviral activity against hepatitis C virus (HCV), indicating upregulation of BST-2 directly participates in IFN-mediated inhibition of HCV production [282]. Zhang’s team also knocked down GP73 in PHHs, demonstrating that GP73 acts as a negative regulator of innate immunity by promoting HCV infection through accelerating degradation of MAVS/TRAF6 [283]. Furthermore, shRNA lentivirus-mediated silencing of PXR significantly inhibited the upregulation of CYP3A4 mRNA, providing evidence that the expression of CYP2E1, CYP1A2, and AhR is regulated by the WNT/β-catenin pathway [284]. Caiment et al. also applied shRNA lentivirus to downregulate the expression of C/EBPα in PHHs, determining that prolonged exposure to VPA (24 ~ 72 h) remarkably reduces the basal and maximal cellular oxygen consumption rates, while C/EBPα, as one of the transcription factors, partially rescues VPA-induced MDF [285].

Since shRNA lentiviral transduction seemingly has no impact on cell viability or liver phenotype, it emerges as an easy-to-use method for long-term or stable downregulation of nuclear receptors and other genes in various cell models, including primary hepatocytes.

### CRISPR-Cas9

CRISPR-Cas9 technology operates by guiding RNA molecules to locate and directly cut or modify the DNA sequence. This enables gene regulation through gene knockout as well as genome editing, including gene insertion or base substitution. Genome editing of hepatocytes is extensively employed to model or intervene in various human liver diseases, encompassing NAFLD, cirrhosis, liver tumors, and other forms of liver injury [286].

The genome-wide analysis is conducted using CRISPR-Cas9 to screen regulatory genes that are either protective or susceptible to APAP-DILI [287]. Additionally, the effect of rifampicin on demethylated and hypermethylated hepatocytes is investigated to explore the relationship between site-specific DNA modifications and DILI [288]. Zhang et al. also found that BACH1 played a central role in aflatoxin B1-induced oxidative damage in hepatocytes by regulating the expression of antioxidant genes based on Genome-Scale CRISPR knockout screening [289]. Interestingly, genetically modified iPSCs edited with CRISPR-Cas9 appear to be well-suited for establishing large-scale DILI screening platforms due to their multiple readouts, high prediction accuracy, and indefinite cell supply. These advantages have been demonstrated for iPSC-HLCs and iPSC-derived HLOs [59, 61].

Compared to gene editing and insertion, the CRISPR-Cas9 system stands out as one of the most mature and efficient methods for gene knockout, so it has also been used to discover the mechanisms of liver toxicity in recent years. Lv et al. suggested that corilagin, a traditional medical drug, attenuated APAP-DILI by enhancing the AMPK/GSK3β-Nrf2 signaling pathway, as evidenced by the knockout of Nrf2 in HepG2 and mice [290]. In the latest study, mice transfected with CRISPR-Cas9-mediated Notch1 knockout or STING activation vectors were employed to highlight the critical role of the macrophage PTEN-NICD/NRF2-STING axis in regulating innate immune responses to APAP-DILI [291].

In summary, it is expected that these three technologies create effective platforms for preclinical drug testing and provide personalized medical support, with the potential to improve drug safety.

## Future perspectives

This review outlines promising in vitro models, cellular responses that may serve as biomarkers, common omics technologies related to DILI, and frequently used molecular technology tools to assess gene function. However, the complexity of its origin and multifactorial nature of DILI, as repeatedly emphasized in this review, currently render the identification of a universal test object for DILI impossible.

The risk of DILI arises from the interplay of both drug and host factors, rather than from either one individually. The diverse metabolism, distribution, and excretion of different drugs in the body vary depending on the activity and rate of different uptake and metabolism pathways, which are influenced by the structure and pharmacokinetic properties of drugs. Drug factors, particularly the concentration of reference drugs, are crucial considerations. However, drug development requires comprehensive consideration of multiple factors, including drug metabolism pathways, pharmacokinetic properties, drug distribution in the body, and toxicity mechanisms. These challenges make it difficult to establish concentration standards for hepatotoxic drugs. Cmax (maximum plasma concentration) seems relatively reasonable, but it overlooks factors like drug accumulation in the liver, protein binding, and the impact of Tmax. Incorporating parameters such as area under the curve (AUC) and half-life offer more comprehensive pharmacokinetic information. Nonetheless, the unification of drug resources, compendiums, and reports remains the most crucial aspect. The Prospective European Drug-Induced Liver Injury Network (PRO-EURO-DILI-NET) Cost Action (*Action CA17112 - COST*) is dedicated to this topic. The alliance was initiated in 2018, with a focus on addressing and resolving advanced developments in preclinical human-relevant models and clinical DILI [292]. Its consensus statement provides detailed guidelines on the requirements for human-based systems to assess hepatotoxicity and to guide future drug safety testing [293].

Regarding host factors, the application of patient-derived cells in DILI modeling seems beneficial in uncovering the influence of individual risk on pathogenesis. The advent of iPSCs enables the development of patient-specific hepatocytes, serving as a host-dependent assay to investigate drug-individual interactions. iPSC-HLCs stand out as a superior alternative to immortalized liver cell lines, positioned as the optimal choice after PHHs (refer to Sect. Hepatocyte-like cells derived from induced pluripotent stem cells). They demonstrate high suitability for personalized cell therapy and screening of candidate pharmacological agents. Addressing concerns regarding their applicability for studying drugs requiring metabolic activation, such as APAP, co-cultures of donor-matched iPSC-differentiated KCs and iPSC-HLCs have been found to possess minimal nonspecific background reactions, effectively detecting inflammation-related drug-induced hepatotoxicity [49]. Moreover, as stated in Sect. Liver organoids, HLOs derived from iPSCs offer tremendous value for performing high-throughput analysis of DILI risk, which is conducive for advancing personalized medicine for DILI.

A novel aspect of DILI research not discussed in this review is the relationship between DILI and the gut microbiota. Gut microbiota can influence the bioavailability, bioactivity, and toxicity of drugs through enzymatic reactions [294]. Additionally, they regulate the expression of host genes involved in metabolic pathways, including nuclear receptor signaling, phase I and II enzymes, and transport proteins [295]. As part of the gut-liver axis, disruptions in gut microbiota activity are believed to contribute to the development of DILI [296]. There seems significant potential for using the gut microbiome as a biomarker to predict the likelihood of developing DILI, as specific microbial signatures may indicate susceptibility to liver toxicity from certain drugs. Overall, the microbiome-DILI connection represents a promising and rapidly evolving area of research, with the potential to improve personalized medicine by predicting DILI risk and optimizing drug safety.

Overall, we believe the ideal system for predicting DILI should consider both the structures and properties of drugs, as well as personalized characteristics of patients, and in silico algorithms can be incorporated to improve accuracy and efficiency. The polygenic risk score (PRS) evaluates an individual’s genetic predisposition to specific traits or diseases, especially in complex conditions influenced by both polygenic and environmental factors [297]. PRS holds great significance for assessing disease risk and promoting precision medicine. Koido et al. pioneered PRS for multi-drug and multi-donor DILI, which has inspired researchers to link in silico protocols with in vitro genomics and transcriptomics. This integration aims to design more specific, efficient, and robust preclinical trials [298]. The combination of two is expected to be the next breakthrough in DILI. Furthermore, the ideal framework for predicting DILI should be based on drug-induced mechanisms that play a causal role and have been validated using adequate genetic perturbation experiments in relevant human cell models.

## Data Availability

This review article does not involve the generation or use of new datasets. All information presented is based onpreviously published studies and publicly available data sources.
